# Evaluation of circulating microRNA profiles in Brazilian women with polycystic ovary syndrome: A preliminary study

**DOI:** 10.1371/journal.pone.0275031

**Published:** 2022-10-07

**Authors:** Giovana De Nardo Maffazioli, Edmund Chada Baracat, José Maria Soares, Kátia Cândido Carvalho, Gustavo Arantes Rosa Maciel

**Affiliations:** 1 Faculdade de Medicina de Sao Paulo, Departamento de Obstetrícia e Ginecologia, Disciplina de Ginecologia, Hospital das Clínicas HCFMUSP, Sao Paulo, SP, Brazil; 2 Laboratório de Ginecologia Estrutural e Molecular (LIM 58), Hospital das Clinicas da Faculdade de Medicina da Universidade de Sao Paulo, Departamento de Obstetricia e Ginecologia, Disciplina de Ginecologia, São Paulo, Brazil; Zhejiang University College of Life Sciences, CHINA

## Abstract

**Objective:**

Polycystic ovary syndrome (PCOS) is a heterogeneous endocrinopathy, which etiology encompasses complex genetic traits associated with epigenetic factors, including differences in microRNA (miRNA) expression in a variety of tissues. The circulating form of these molecules is raising attention in the syndrome not only as potential biomarkers of PCOS but also as possible therapeutic targets. The aim of this study was to explore the circulating miRNA profiles present in a cohort of Brazilian women with and without PCOS and to evaluate the potential role of miRNAs in the pathophysiology of the syndrome.

**Methods:**

Cross-sectional study of 36 well-characterized PCOS women and 16 healthy controls. Clinical, hormone and metabolic data were recorded and evaluated. The expression profile of the 201 circulating miRNA selected were analyzed by taqman quantitative real time polymerase chain reactions (RT-PCR) using a customized Open Array platform. Statistical and bioinformatic analyzed were performed.

**Results:**

Circulating *miR-21-5p*, *miR-23a-3p* and *miR-26a-5p* were upregulated, and *miR-103a-3p*, *miR-376a-3p*, *miR-19b-3p* and *miR-222-3p* were downregulated in women with PCOS compared to healthy normo-ovulatory controls. *miR-21-5p*, *miR-103a-3p* and *miR-376a-3p* levels correlated positively with androgen levels. These miRNAs, in combination, were related to pathways involved in insulin signaling, steroids biosynthesis and endothelial regulation as well as in folliculogenesis.

**Conclusion:**

In this study, we identified a specific circulating miRNA signature in Brazilian women with PCOS. According to our data, circulating *miR-21-5p*, *miR-23a-3p*, *miR-26a-5p*, *miR-103a-3p*, *miR-376a-3p*, *miR-19b-3p* and *miR-222-3p* may represent potential candidates for differential diagnosis of PCOS in the future.

## Introduction

Polycystic ovary syndrome (PCOS) is a complex endocrinopathy that affects between 5% and 17% of women of reproductive age, depending on the population studied and the diagnostic criteria used [1–5]. It is characterized by chronic anovulation, hyperandrogenism, and the presence of polycystic ovaries on ultrasound [6,7], and it is frequently associated with infertility, insulin resistance, obesity and an increased risk of metabolic diseases such as type 2 diabetes mellitus, metabolic syndrome, cardiovascular disease and dyslipidemia [8–10].

The etiologies of the syndrome and its comorbidities are not fully understood, but they are known to have a complex genetic component with a high degree of heritability [11,12] and to be associated with epigenetic changes [13–15]. Thus, a greater understanding of the molecular mechanisms underlying the pathophysiology of the syndrome is needed and might lead to the identification of novel diagnostic and therapeutic targets.

MicroRNAs (miRNAs) are small noncoding RNAs that are produced in all the cells of the body; they are associated with the posttranscriptional epigenetic regulation of gene expression [16] and are involved in the maintenance of metabolic homeostasis [17], obesity and diabetes [17]. The study of miRNAs in women with PCOS has aroused great interest in recent years. Most of these studies have focused on analysis of differential expression of miRNAs in women with and without PCOS using various tissues (serum, adipocytes, follicular fluid, and ovarian cells) [18–22].

Currently, approximately 79 miRNAs have been identified as showing differential expression [23] in PCOS, and the expression profiles of these miRNAs have been related to their varied characteristics and comorbidities. However, the results obtained in different studies vary, and the role that these molecules play as markers or mediators of PCOS requires further elucidation. Some possible explanations for the divergences in the results obtained in the existing studies include insufficient sample size, variation in ethnicity, age, body mass index (BMI), inclusion criteria or comparison parameters, as well as differences in the technical aspects of the studies such as the type of analysis performed or the cutoff points used to define differential expression [23].

Due to the contrasting results and the potential role that epigenetic changes may play in the pathophysiology of PCOS, it is important that more detailed studies be conducted to replicate the findings reported in other populations and to make it possible to understand the mechanisms that these or other miRNAs may play in the syndrome and in its associated comorbidities. Thus, the aim of this study was to explore circulating miRNA profiles in a cohort of Brazilian women with and without PCOS. The Brazilian population is characterized by racial diversity and wide miscegenation, characteristics that may result in the presence of unique epigenetic traits.

## Material and methods

### Study population and setting

This cross-sectional study included 38 participants with PCOS who were selected from among the patients who attended their first clinical appointments between 2016 and 2018 at the Division of Ginecologia Endocrina da Disciplina de Ginecologia do Hospital das Clinicas da Faculdade de Medicina da Universidade de Sao Paulo, Brazil (HC-FMUSP). These patients complained of hyperandrogenism and/or menstrual disturbances and met the criteria for the diagnosis of PCOS [7]. The 21 participants in the control group were recruited from the general population by convenience sampling. All participants received guidance on the research in accordance with the Declaration of Helsinki and signed a Free and Informed Consent Form (ICF). This project was approved by the Ethics Committee for the Analysis of Research Projects of the Clinical Directorate of HC-FMUSP (number 2.622.258).

All control participants were healthy and had regular menstrual cycles (between 24 and 38 days in length) and hormonal profiles within the normal range for the age group. All participants with PCOS met the diagnostic criteria listed in the Rotterdam consensus [7]. This diagnostic criterion was chosen for the classification of participants with PCOS since it has been widely used in clinical studies [24].

The recruited participants were interviewed and subjected to detailed physical examination according to the clinical protocol. Individuals with diabetes mellitus, any type of cancer, coronary artery disease, stroke, liver cirrhosis, thyroid diseases or a history of autoimmune disease were excluded. Women who had been pregnant, had an abortion or undergone a surgical procedure during the year prior to the study were excluded. Women who had used medications that alter the hormonal and metabolic profile, including hormonal contraceptives, hypoglycemic agents, statins/fibrates and glucocorticoids, during the 3 months prior to the study were also excluded. Gynecological and obstetric history as well as family history were collected.

The data collected during the physical examination included weight, height, body mass index (BMI), waist circumference (WC), systemic blood pressure, and assessment of the presence of acne, alopecia, voice deepening, clitoral enlargement and acanthosis nigricans. WC measurement was performed according to the standards recommended by the World Health Organization (WHO) [25]. Evaluation of the presence of clinical hyperandrogenism (hirsutism) was performed using the Ferriman-Gallwey scale modified by Hatch [26].

### Biochemical analyses

Peripheral blood samples were collected from all participants after the medical interview and used to assess their hormonal and metabolic profiles. The blood samples were obtained after the subjects had fasted for 12 hours and during the early follicular phase of the menstrual cycle if the participants had regular menstrual cycles or at any stage of the cycle in the case of participants who had oligo/amenorrhea. An oral glucose tolerance test 2 hours after ingestion of 75 g of glucose was also performed in the PCOS group. The reference values ​​for the hormonal and metabolic parameters were obtained through the core laboratory of HC-FMUSP.

### Transvaginal ultrasonography

To assess the polycystic ovarian pattern, transvaginal ultrasonography was performed on the participants in the PCOS group. According to the Rotterdam consensus, ovaries were classified as a polycystic appearance if they met at least one of the following criteria: twelve or more follicles smaller than 10 mm in at least one ovary and/or ovarian volume greater than 10 cm^3^ in at least one ovary [24].

### Blood collection, microRNA selection and sample quality control

Peripheral blood was collected by venipuncture of the forearm and stored in 5-mL BD Vacutainer® tubes containing clot activator, which accelerates the clotting process, and separator gel to obtain serum of the highest quality. After collection, the samples were homogenized manually, and the tubes were kept in a vertical position for 30 minutes at room temperature to retain the clot. The tubes were then subjected to centrifugation at 2,000 x g at 4°C for 15 minutes using a Sorvall ST 16R centrifuge (ThermoFisher Scientific, Waltham, MA, USA). The serum was transferred to 2-mL DNase/RNase-free tubes and stored in 1-mL aliquots at -80°C until RNA extraction.

A total of 201 miRNAs that have been reported in the literature to be related to PCOS or to the expression of genes related to insulin metabolism, pancreatic β cell function or type 2 diabetes mellitus were selected for study [23,27–30]. The FirePlex Discovery Engine platform (available at www.fireflybio.com) was used as an aid in their selection. To avoid contamination with miRNA from blood cells, only samples without hemolysis that showed an absorbance of ≤0.2 on the Nanodrop 2000 spectrophotometer (Thermo Scientific, Waltham, MA, USA) were considered adequate for the extraction of genetic material [31].

### MicroRNA extraction and preparation

The samples were thawed on ice and then centrifuged for 5 minutes at 1,6000 x g at a temperature of 4°C (Sorvall Legend Micro 17R, Thermo Scientific) to remove cryoprecipitates. Soon after, 200 μL of the supernatant was transferred to a new 2-mL microtube. The total RNA extraction process was conducted using the miRNeasy Serum/Plasma kit (Qiagen, Germany) according to the manufacturer’s instructions.

To monitor the efficiency of total RNA extraction, 0.02 pmol/μl synthetic miRNA-39-3p (*Caenorhabditis elegans—cel-miR-39-3p*; Thermo Scientific) was added to the sample during the extraction process, as recommended by the manufacturer.

The extracted RNA was eluted with 14 μL of nuclease-free water (Qiagen, Germany). The concentration and purity of the RNA were assessed using a spectrophotometer (Nanodrop 2000, Thermo Scientific). Due to the low concentration of circulating miRNA in serum, cDNA synthesis was performed using the Taqman® Advanced miRNA Assay kit for low-throughput samples (Thermo Scientific) according to the protocol provided by the manufacturer.

### Analysis of MicroRNA gene expression

Real-time PCR was performed to test the amplification of the 201 miRNAs of interest using the Open Array platform (Custom-Configured TaqMan*®* Open Array^TM^ Plates, Thermo Scientific). The reactions were performed in a final volume of 25.0 μL using Taqman Open Array Real-Time PCR Master Mix (Taqman® Advanced miRNA Assay, Thermo Scientific) and were transferred to open array chips in an automated manner using the AccuFill*®* device (Thermo Scientific). The Open Array chips were then sealed and incubated in the QuantStudio 12K Flex Real-Time PCR System (Thermo Scientific) for RT–PCR.

The amplification curves for each miRNA were analyzed using ThermoFisher Cloud software (https://www.thermofisher.com/account-center/cloud-signin-identifier.html). miRNAs that did not present adequate amplification profiles based on these curves were excluded according to the algorithm present in the software itself, as indicated by the manufacturer. At the conclusion of this analysis, 97 miRNAs remained. Of these, 3 miRNAs (*let-7b-5p*, *miR486-5p* and *miR-92a-3p*) were selected as endogenous controls. These miRNAs were selected, as recommended by the reagent manufacturer (Thermo Scientific), because they showed the most homogeneous amplification curves in all samples among all evaluated miRNAs, as determined from their amplification scores (≤1.5). The software considered the mean of the comparative threshold cycle (CT) values of these three genes when calculating the relative expression (ΔCT) of the other miRNAs. The ΔCT was calculated by subtracting the CT from the PCOS group or the control group from the average of the CT values of the endogenous controls (*let-7b-5p*, *miR486-5p* and *miR-92a-3p*).

Expression values were calculated using the ΔΔCT method and are presented as relative quantification (RQ) and fold regulation (FR). Values were obtained as described by Calvano et al. [32]. The cutoff expression values of + 1.0 and -1.0, based on reference values of expression, were used to identify the miRNAs that were hyperexpressed (1) and hypoexpressed (-1) in the PCOS group compared to the control group.

### Interaction network analysis of the miRNAs and genes associated with PCOS and metabolic dysfunctions

To better understand the roles of the selected miRNAs with the most significant differential expression profiles between the PCOS group and the control group in the pathophysiology of PCOS, the interaction networks of these miRNAs with their potential targets were evaluated separately and together using the miRTargetLink Human platform (https://ccb-web.cs.uni-saarland.de/mirtargetlink/).

Subsequently, a gene ontology (GO) biological process and molecular function enrichment analysis was performed in relation to the interaction network of the genes targeted by at least two differentially expressed miRNAs using the GeneNetwork platform, available at https://genenetwork.nl/. Enrichment analysis of the main signaling pathways related to these genes was performed using the same platform. The GeneNetwork platform integrates data from the Kyoto Encyclopedia of Genes and Genomes (KEGG) and from the REACTOME pathways database. The raw data were then manually screened, and the biological and molecular information as well as information on the enriched signaling pathways, which may be involved in the pathophysiology of PCOS, were selected.

### Statistical analysis

Analysis of the collected data was conducted using the STATA program (version 14.0). The graphs were prepared using GraphPad Prism (version 5.0). The data are presented as mean ± standard deviation, median (interquartile range) or percentage depending on the nature of the data and were checked for normality using distribution graphs and the Shapiro–Wilk test. For the analysis of differences between two groups, Student’s t test or Wilcoxon’s rank sum test was used according to normality for continuous variables. For the analysis of categorical variables, the chi-square test or Fisher’s exact test (when more appropriate) was used. As expected, the control participants had significantly lower BMI than did the participants with PCOS. Because of this, the P values of the laboratory and miRNA expression data were adjusted using covariance analysis (ANCOVA). Spearman’s rank correlation was used to relate the miRNA expression profiles to clinical and laboratory characteristics. Sample calculation was based on a 50% difference in the measures of relative expression of miRNAs between the PCOS and control groups, with a common standard deviation of 0.5, according to a previous study [33]. A sample of 17 participants per group showed 80% power with an α of 0.05. To make it possible to conduct an exploratory analysis of subgroups within the SOP, it was decided to recruit twice as many participants with PCOS as participants without PCOS.

## Results

### Clinical characteristics

A total of 58 women were recruited; 38 of these were in the PCOS group, and 21 were in the control group. After samples with insufficient amounts of genetic material or quality of amplification in RT–PCR were excluded, 36 women with PCOS and 16 controls remained. Analysis of the clinical characteristics of these women showed that the two groups were adequately matched in terms of age (p = 0.16) and ethnicity (p = 0.87). As expected, participants with PCOS had higher BMI (p = 0.03), WC (p = 0.001), higher incidence of acanthosis nigricans (p = 0.02) and significantly different Ferriman-Gallwey scores (p = 0.0001) in relation to the control group [Table 1].

**Table 1:** Clinical, hormonal and metabolic characteristics of women with PCOS versus controls.

	PCOS (n = 36)	Control (n = 16)	P-value	P-value****
Clinical Characteristics	*Mean±SD*	*Mean±SD*		
Age (years)	28.9±4.9	26.8±5.1	0.16	-
Ethnicity (%) Caucasian Afro-Brazilian Mix	37.1 17.1 45.7	43.8 12.5 43.8	0.87**	-
BMI (m/kg^2^)	32.3±7.6	26.9±8.9	**0.03**	-
WC (cm)	102.5±17.4	84.3±18.6	**0.001**	-
Acanthosis nigricans (%)	38.9	6.3	**0.02** ***	-
Ferriman-Gallwey modified (score)	7.0 (5–12)	1.5 (0–4)	**0.0001** *	-
**Transvaginal Ultrasound**	*Median (IQR)*	*Median (IQR)*		-
Right ovary volume (cm^3^)	12.0 (9,5–15,4)	-	-	-
Left ovary volume (cm^3^)	11.9(8,0–15,5)	-	-	-
Polycystic pattern (%)	66.7	-	-	-
**Hormonal/Metabolic Characteristics**	*Mean±SD*	*Mean±SD*		
FSH (IU/L)	5.4±1.9	6.5±1.3	**0.04**	0.19
LH (IU/L)	12.9±6.4	7.0±5.5	**0.0001** *	**0.001**
LH/FSH	2.47±0.98	1.10±0.89	**<0.0001** *	**<0.0001**
Total testosterone (ng/dL)	53±25	34±11	**0.003** *	**0.02**
Free testosterone (pmol/L)	33±18	14±7	**<0.0001** *	**0.003**
SHBG (nmol/L)	41.8±24.7	62.4±29.5	**0.01** *	0.10
AMH (ng/mL)	7.48±4.16	2.89±2.05	**<0.0001**	**<0.0001**
Fasting glucose (mg/dL)	89.1±12.0	82.5±8.4	0.07*	0.30
Fasting glucose/insulin	5.3±3.7	10.9±5.1	**0.0001** *	**0.001**
HOMA-IR	6.9±7.6	2.0±1.1	**0.0001** *	0.10
Total Cholesterol (mg/dL)	182±39	190±37	0.48	0.48
LDL-C (mg/dL)	104±32	103±35	0.92	0.79
HDL-C (mg/dL)	52±20	65±20	**0.004** *	0.15
Triglycerides (mg/dL)	140±99	77±35	**0.01** *	**0.046**

BMI: Body mass index; WC: Waist circumference; FSH: Follicular stimulating hormone; LH: Luteinizing hormone; SHBG: Sexual hormone binding globulin; AMH: Antimullerian hormone; HOMA-IR: Homeostatic model assessment for insulin resistance; LDL-C: Low density lipoprotein—cholesterol; HDL-C high density lipoprotein–cholesterol.

Continuous data reported as mean±standard deviation or median (interquartile range); categorical data reported as percentage (%).

Student T-test used for the analysis of normal distributed continuous data.

*Wilcoxon rank-sum test used for the analysis of non-normal distributed continuous data.

** Chi-squared test used for the analysis of categorical data.

*** Fisher Exact Test.

****p-value after controlling for BMI.

**Reference values:** FSH: 3.5–12.5 IU/L; LH: 2.4–12.6 IU/L; Total testosterone: <49 ng/dL; Free testosterone: 2.4–37.0 pmol/L; SHBG: 32.2–128.0 nmol/L; AMH: 1.2 a 3.4 ng/mL; Fasting glucose: 70–99 mg/dL; HOMA-IR: <2.7; Total Cholesterol: <200 mg/dL; LDL-C: <160 mg/dL; HDL-C: > 60 mg/dL; Triglycerides: <150 mg/dL.

Among women with PCOS, 97.2% had oligo/amenorrhea, 68.7% had clinical and/or laboratory hyperandrogenism, and 77.2% had increased ovarian volume or a polycystic pattern on ultrasound. Of the participants who reported menstrual changes, 25.7% had amenorrhea, and 74.3% reported oligo/amenorrheic cycles.

### Hormonal and metabolic characteristics

The hormonal characteristics of the two groups are described in Table 1. Women with PCOS had higher mean concentrations of LH (12.9 ± 6.4 IU/L vs. 7.0 ± 5.5 IU/L, p = 0.001), higher LH/FSH ratios (2.47 ± 0.98 vs. 1.10 ± 0.89, p <0.0001), higher total testosterone (53 ± 25 ng/dL vs. 34 ± 11 ng/dL, p = 0.003) and free testosterone (33 ± 18 pmol/L vs. 14 ± 5, p <0.0001) levels, and higher AMH (7.48 ± 4.16 ng/mL vs. 2.89 ± 2.05 ng/mL, p <0.0001) than the controls, even after adjustment for BMI. In contrast, mean SHBG concentrations were higher in the control participants than in those with PCOS (62.4 ± 29.5 nmol/L vs. 41.8 ± 24.7 nmol/L, p = 0.01), maintaining a trend after adjustment for BMI. FSH showed no statistically significant difference between the two groups.

Regarding the analysis of metabolic profiles [Table 1], women with PCOS had higher mean fasting glucose/insulin ratios (5.3±3.7 vs. 10.9±5.1, p = 0.0001), higher HOMA-IR (6.9±7.6 vs. 2.0±1.1, p = 0.001), and higher triglyceride levels (140±99 mg/dL vs. 77±35 mg/dL, p = 0.01) than controls, even after adjustment for BMI, and HOMA-IR maintained a trend after this adjustment. Although participants with PCOS also had lower mean HDL-C levels than controls, these differences did not persist after adjustment for BMI. The two groups did not differ in fasting glucose or total or LDL cholesterol levels.

### Differences in circulating miRNA expression profiles between PCOS and controls

Of the 201 miRNAs analyzed, 103 could not be assessed due to the absence of expression in the target or control group samples. *Let-7b-5p*, *miR-486-5p and miR92a-5p* were selected as endogenous controls because they presented adequate stability patterns among all samples. A total of 95 miRNAs were included in the differential expression analysis in which the two groups were compared. Only 33 of these miRNAs showed differential expression profiles/values (4 were upregulated, and 29 were downregulated) in the PCOS group compared to controls [Table 2]. For these miRNAs, the mean differences in relative expression between the two groups were analyzed. To verify the statistical relevance of the findings and the adjustment for BMI, the remaining 15 miRNAs that showed significant differences in expression between the two groups (*miR-21-5p*, *miR-23a-3p* and *miR-26a-5p* (overexpressed in the PCOS group) and *miR-103a-3p*, *miR-361-5p*, *miR376a-3p*, *miR-382-5p*, *miR-128-3p*, *miR181b-5p*, *miR-19b-3p*, *miR-21-3p*, *miR-222-3p*, *miR-33a-5p*, *miR-494-3p*, and *miR-885-5p* (hipoexpressed in PCOS group) were analyzed [Table 3]. To decrease the chance of bias in our findings, only miRNAs that presented amplification in more than 80% of the study samples (*miR-103a-3p*, *miR-21-5p; miR-23a-3p*, *miR-26a-5p*, *miR-376a-3p*, *miR-19b-3p and miR-222-3p*) were chosen for correlation and network interaction analysis.

**Table 2:** Fold regulation (FR*)* of the 95 evaluated miRNAs in the PCOS group in relation to controls.

miRNA	Fold Regulation (n)	miRNA	Fold Regulation (n)	miRNA	Fold Regulation (n)
Let-7e-5p	**-1.593 (8** *)*	miR-26a-5p	**1.091 (36)**	miR-151a-5p	-0.452 (33)
miR-143-3p	-0.826 (19)	miR-26b-5p	0.214 (35)	miR-15a-5p	-0.466 (36)
miR-335-5p	0.655 (12)	miR-27a-3p	0.605± (34)	miR-16-5p	0.479 (36)
Let-7a-5p	0.418 (30)	miR-27b-3p	-0.534 (30)	miR-17-5p	0.208 (35)
Let-7c-5p	0.943 (7)	miR-296-5p	-0.483 (15)	miR-181a-5p	-0.635 (36)
Let-7d-3p	**-1.345 (12** *)*	miR-29a-3p	0.240 (35)	miR-181b-5p	**-1.601 (32)**
Let-7f-5p	0.689 (30)	miR-29b-3p	-0.684 (31)	miR-18a-3p	**-1.585 (19)**
Let-7g-5p	0.445 (36)	miR-29c-3p	**-2.006 (9)**	miR-18a-5p	-0.093 (33)
Let-7i-5p	-0.485 (36)	miR-301a-3p	**-1.653 (18)**	miR-191-5p	0.267 (36)
miR-103a-3p	**-1.062 (34** *)*	miR-30c-5p	0.036 (31)	miR-192-5p	**-1.364 (15)**
miR-107	-0.561 (36)	miR-30d-5p	-0.005 (36)	miR-197-3p	**1.979 (11)**
miR-125a-5p	-0.711 (35)	miR-320b	-0.394 (21)	miR-199a-3p	**-1.488 (20)**
miR-125b-5p	-0.909 (36)	miR-324-3p	**4.378 (11)**	miR-19b-3p	**-1.717 (33)**
miR-126-5p	-0.002 (34)	miR-324-5p	-0.552 (10)	miR-20a-5p	0.152 (36)
miR-127-3p	**-2.503 (13)**	miR-361-5p	**-1.634 (31)**	miR-21-3p	**-1.404 (10)**
miR-130a-3p	-0.825 (30)	miR-376a-3p	**-1.200 (36)**	miR-222-3p	**-1.241 (35)**
miR-130b-3p	-0.602 (11)	miR-382-5p	**-2.257 (13)**	miR-223-3p	0.323 (36)
miR-133a-3p	-0.995 (30)	miR-424-5p	-0.751 (29)	miR-28-3p	**-1.232 (26** *)*
miR-146a-5p	-0.590 (36)	miR-433-3p	**-2.061 (14)**	miR-30b-5p	0.228 (23)
miR-148a-3p	-0.708 (33)	miR-451a	-0.967 (36)	miR-320a-3p	-0.300 (35)
miR-150-5p	-0.565 (36)	miR-7-5p	0.311 (20)	miR-33a-5p	**-1.622 (17)**
miR-15b-5p	0.639 (6)	miR-99b-5p	-0.928 (23)	miR-342-3p	-0.735 (36)
miR-185-5p	0.869 (36)	miR-103a-25p	0.056 (17)	miR-483-5p	**-1.575 (7)**
miR-190a-5p	0.155 (12)	miR-106b-3p	-0.902 (28)	miR-494-3p	**-3.977 (13)**
miR-194-5p	-0.717 (23)	miR-10b-5p	-0.545 (24)	miR-505-3p	**-2.357 (26)**
miR-195-5p	**-1.055 (12)**	miR-122-5p	-0.854 (35)	miR-584-5p	**-1.136 (11)**
miR-199a-5p	**-1.488 (20** *)*	miR-126-3p	0.017 (35)	miR-660-5p	**-3.002 (22)**
miR-19a-3p	0.367 (34)	miR-128-3p	**-1.982 (29)**	miR-885-5p	**-2.020 (22)**
miR-21-5p	**1.098 (36)**	miR-132-3p	-0.874 (9)	miR-92b-3p	0.376 (36)
miR-23a-3p	**-1.506 (36)**	miR-144-5p	**-2.865 (4)**	miR-93-5p	-0.027 (35)
miR-23b-3p	-0.796 (24)	miR-145-5p	-0.126 (33)	miR-101-3p	-0.294 (22)
miR-24-3p	-0.024 (36)	miR-148b-3p	-0.244 (31)		

Data represented as the mean of the PCOS samples.

Fold regulation cut-off values were stablished at >1 for hiperexpression and at <1 por hipoexpression (bold).

The number between parenthesis indicates the number of samples in which the miRNA showed amplification.

**Table 3:** Comparison of the expression values of the 33 miRNAs selected between PCOS and controls.

miRNA	PCOS	Control	p-value	P-value**
	*Mean±SD (n)*	*Mean±SD (n)*		
Let-7e-5p	0.031±0.016 (9)	0.065±0.24 (3)	**0.03** *	0.37
Let-7d-3p	0.353±0.258 (12)	0.477±0.187 (3)	0.56*	0.28
miR-103a-3p	0.021±0.016 (34)	0.033±0.016 (13)	**0.01** *	**0.04**
miR127-3p	0.005±0.004 (13)	0.015±0.013 (4)	0.05*	0.05
miR-199a-5p	0.028±0.030 (20)	0.065±0.053 (4)	0.22*	0.09
miR-21-5p	0.128±0.080 (36)	0.085±0.070 (16)	**0.04**	**0.045**
miR-23a-3p	0.166±0.171 (36)	0.041±0.254 (16)	**0.0001** *	**<0.0001**
miR-26a-5p	0.692±0.465 (36)	0.396±0.250 (16)	**0.02**	**0.03**
miR-29c-3p	0.101±0.095 (9)	0.224±0.066 (2)	0.09*	0.15
miR-301a-3p	0.007±0.008 (18)	0.013±0.005 (3)	0.16*	0.08
miR-324-3p	0.017±0.027 (11)	0.0002 (1)	0.19*	0.50
miR-361-5p	0.010±0.009 (31)	0.020±0.012 (7)	**0.02** *	**0.03**
miR-376a-3p	0.008±0.009 (36)	0.015±0.006 (14)	**0.0004** *	**0.01**
miR-382-5p	0.003±0.003 (13)	0.015±0.011 (4)	**0.02** *	**0.02**
miR-433-3p	0.016±0.013 (14)	0.052±0.049 (4)	0.06*	0.003
miR-128-3p	0.002±0.002 (29)	0.008±0.005 (5)	**0.003** *	**0.001**
miR-144-5p	0.041±0.070 (4)	0.124±0.093 (7)	0.06*	0.56
miR-181b-5p	0.014±0.021 (32)	0.043±0.051(9)	**0.04** *	**0.01**
miR-18a-3p	0.006±0.015 (18)	0.008±0.0002 (2)	0.08*	0.75
miR-192-5p	0.200±0.226 (15)	0.335±0.149 (3)	0.21*	0.36
miR-197-3p	13.237±10.303 (11)	2.677±1.564 (6)	**0.02** *	0.06
miR-199a-3p	0.279±0.030 (20)	0.065±0.053 (4)	0.22*	0.09
miR-19b-3p	0.186±0.171 (34)	1.088±1.046 (8)	**0.0001** *	**<0.001**
miR-21-3p	0.016±0.008 (10)	0.038 (1)	0.11*	**0.001**
miR-222-3p	0.010±0.010 (35)	0.018±0.011 (15)	**0.003** *	**0.01**
miR-28-3p	0.013±0.013 (26)	0.020±0.015 (7)	0.12*	0.11
miR-33a-5p	0.027±0.022 (17)	0.066±0.027 (3)	**0.04** *	**0.02**
miR-483-5p	0.021±0.018 (7)	0.022 (1)	0.83*	0.70
miR-494-3p	0.004±0.005 (13)	0.048 (1)	0.11*	**<0.001**
miR-505-3p	0.007±0.009 (26)	0.012±0.006 (4)	0.10*	0.37
miR-584-5p	1.482±1.324 (11)	0.978±0.458 (4)	0.79*	0.50
miR-660-5p	0.008±0.019 (22)	0.011±0.004 (2)	0.14*	0.73
miR-885-5p	0.009±0.010 (22)	0.023±0.012 (3)	0.05*	**0.01**

Expression Values (2^-ΔCT^) normalized by the constitutive/housekeeping/endogenous miRNAs.

Data reported as mean±standard deviation.

Student T-test used for the analysis of normal distributed continuous data.

*Wilcoxon rank-sum test used for the analysis of non-normal distributed continuous data.

**p-value after controlling for BMI.

### Correlations between clinical and laboratorial characteristics and circulating miRNA expression in women with PCOS

Correlation coefficients were obtained to determine whether the seven miRNAs identified as having differential expression in the PCOS group were related to any particular clinical and/or laboratory features of the syndrome. As depicted in Table 4, *miR-103a-3p*, *miR-21-5p* and *miR-376a-3p* were positively correlated with total testosterone level (rho = 0.39, p = 0.02; rho = 0.48, p = 0.0.004; and rho = 0.34, p = 0.047, respectively). Other relevant characteristics were not correlated with these miRNAs in our PCOS group.

**Table 4:** Correlations between clinical and laboratorial characteristics with miRNAs in PCOS women.

	miR103a-3p		miR-21-5p		miR-23a-3p		miR-26a-5p		miR-376a-3p		miR-19b-3p		miR-222-3p	
	rho	p	rho	P	rho	p	rho	p	rho	p	rho	p	rho	p
**BMI**	0.15	0.42	-0.02	0.92	0.18	0.31	-0.004	0.98	0.05	0.76	0.24	0.18	-0.22	0.24
**LH/FSH**	-0.20	0.26	-0.11	0.53	0.03	0.88	-0.12	0.52	-0.17	0.33	-0.06	0.75	0.30	0.10
**Total testosterone**	0.39	**0.02**	0.48	**0.004**	0.24	0.17	0.31	0.08	0.34	**0.047**	0.18	0.32	0.25	0.15
**Free testosterone**	0.23	0.21	0.21	0.21	0.18	0.32	0.17	0.33	0.24	0.16	0.21	0.24	0.04	0.85
**SHBG**	0.16	0.38	0.26	0.14	0.06	0.74	0.17	0.33	0.10	0.56	-0.14	0.43	0.30	0.10
**AMH**	0.15	0.43	0.17	0.35	0.14	0.43	0.11	0.56	0.11	0.53	-0.06	0.73	0.27	0.13
**HOMA-IR**	-0.01	0.95	-0.09	0.63	-0.001	0.99	-0.02	0.92	-0.05	0.79	0.16	0.38	-0.25	0.17
**Fasting Glucose**	0.12	0.50	-0.08	0.66	0.04	0.83	-0.04	0.85	-0.02	0.91	0.08	0.67	-0.28	0.11
**Glucose OGTT**	0.04	0.85	-0.13	0.48	0.15	0.42	0.10	0.61	0.08	0.66	0.19	0.30	-0.14	0.44

BMI: Body mass index; FSH: Follicular stimulating hormone; LH: Luteinizing hormone; SHBG: Sexual hormone binding globulin; AMH: Antimullerian hormone; HOMA-IR: Homeostatic model assessment for insulin resistance; OGTT: Oral glucose intolerance test.

Spearman’s rank correlations.

### Interaction networks and enrichment analysis for miR-103a-3p, miR-21-5p, miR-23a-3p, miR-26a-5p, miR-376a-3p, miR-19b-3p and miR-222-3p and their main molecular targets involved in the development of PCOS and its associated metabolic dysfunctions

**Fig 1:**
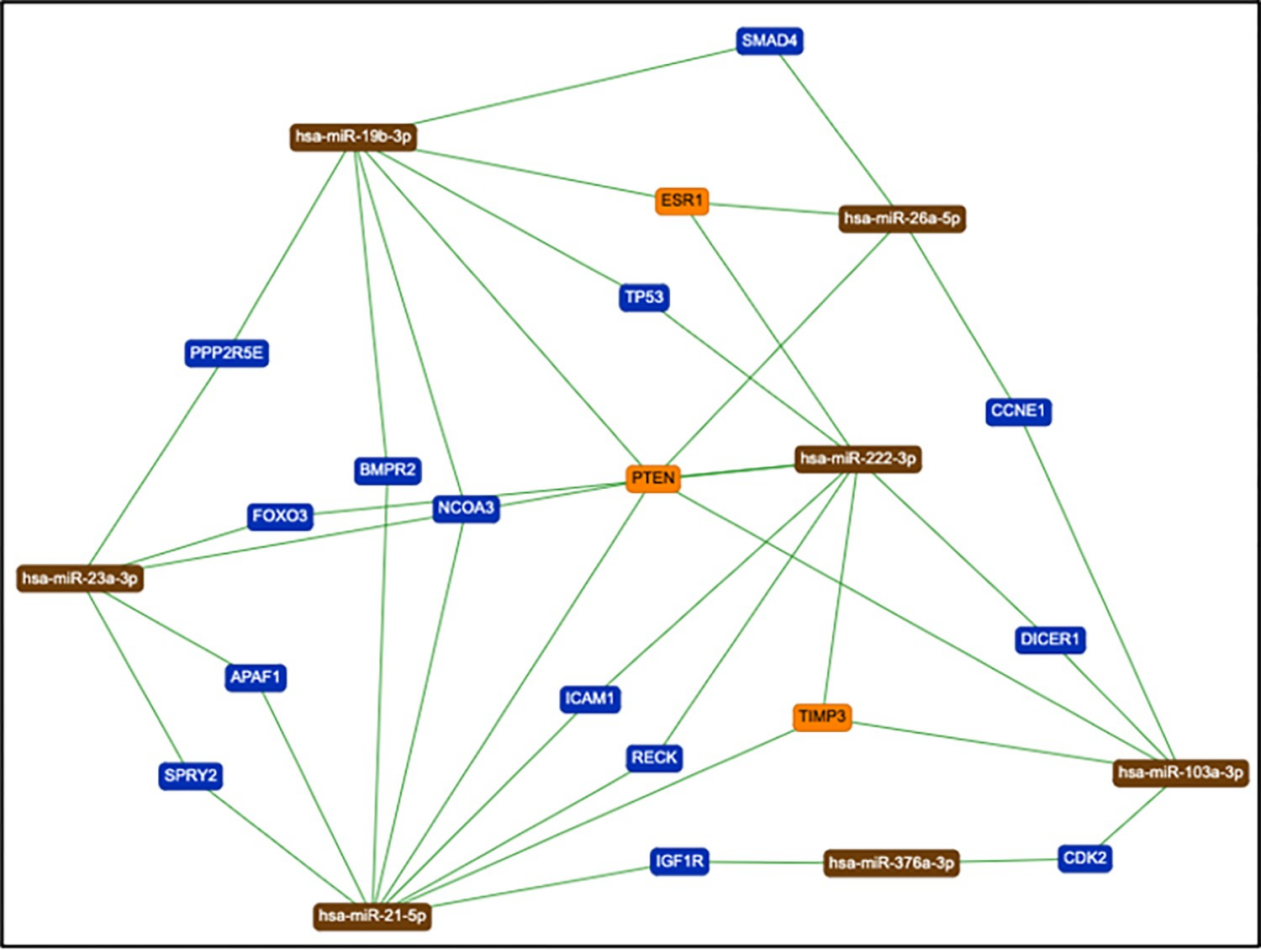
Mapping the target genes (n = 17) that were common to at least two miRNAs with differential expression in the PCOS groups.

An interaction network of potential target genes related to each miRNA was constructed. Including only interactions for which there is support in the literature, *miR-103a-3p* was predicted to interact with 16 target genes, and *miR-21-5p*, *miR-23a-3p*, *miR-26a-5p*, *miR-376-3p*, *miR-19b-3p*, and *miR-222-3p* were predicted to interact with 99, 32, 52, 10, 24, and 32 genes, respectively. Of those genes, 17 were related to at least two of these miRNAs [Fig 1].

Subsequently, enrichment analysis of biological processes and molecular functions in relation to the genetic ontology (GO) of these target genes was performed. Table 5 shows a selection of the main biological processes and molecular functions of the 17 genes that are related to at least two of the miRNAs that were found to be differentially expressed in the PCOS patients.

**Table 5:** Genetic ontology (GO) of biologic process and function and KEGG and REACTOME data banks pathway analysis associated to the seventeen target genes related to at least two miRNAs.

**Biologic Processes**	**p-value**	**Molecular Functions**	**p-value**
Intrinsic apoptotic signaling pathway in response to damage by p53 class mediator	4.2x10^-6^	Protein tyrosine/serine/threonine phosphatase activity	1.7x10^-4^
Epidermal growth factor receptor signaling pathway	1.6x10^-4^	SMAD binding	1.9x10^-3^
Positive regulation of fat cell differentiation	5.4x10^-4^	Protein serine/threonine kinase activity	2.8x10^-3^
Non-canonical Wnt signaling pathway	1.3x10^-3^	TGF-*β* binding	5.1x10^-3^
Cellular response to TGF-*β* stimulus	1.5x10^-3^	Neuropeptide hormone activity	5.5x10^-3^
Regulation of apoptotic process	2.5x10^-3^	Protein tyrosine kinase activity	8.6x10^-3^
Pathway-restricted SMAD protein phosphorylation	4.6x10^-3^	Protein serine/threonine/tyrosine kinase activity	0.01
TGF-*β* signaling pathway	7.3x10^-3^	G-protein coupled receptor activity	0.02
Retinoic acid metabolic process	8.1x10^-3^	Phosphatidylinositol-3-phosphatase activity	0.03
Regulation of cyclin-dependent protein serine/threonine kinase activity	0.01	Wnt-activated receptor activity	0.03
Positive regulation of inflammatory response	0.01	Phosphatidylinositol 3-kinase binding	0.03
Activation of MAPK activity	0.02		
Positive regulation of angiogenesis	0.02		
VEGF signaling pathway	0.02		
**KEGG Pathways**	**p-value**	**REACTOME Pathways**	**p-value**
Insulin signaling pathway	5.5x10^-4^	Transcriptional activity of SMAD2/SMAD3:SMAD4 heterotrimer	1.7x10^-5^
Steroid hormone biosynthesis	2.2x10^-3^	Signaling TGF-*β* Family members	4.1x10^-5^
Erbb signaling pathway	3.6x10^-3^	Signaling by receptor tyrosine kinases	2.0x10^-4^
Retinol metabolism	3.9x10^-3^	Signaling by VEGF	1.9x10^-3^
MAPK signaling pathway	5.7x10^-3^	MAP2K and MAPK activation	2.9x10^-3^
Inositol phosphate metabolism	6.2x10^-3^	Negative regulation of MAPK pathway	0.01
TGF-*β* signaling pathway	9.6x10^-3^	TGF-*β* receptor signaling activates SMADs	0.02
Adipocytokine signaling pathway	0.03	Insulin receptor recycling	0.04

Biologic Process and Functions and Pathways described were selected based on their potential to be related to PCOS physiopathology.

To obtain a list of signaling pathways that are likely controlled by these genes, the main interaction pathways that are overrepresented in the analysis and that may be related to the pathophysiology of PCOS were selected; these are listed in Table 5.

## Discussion

For this study, we selected 201 circulating miRNAs with potential contribution to the pathophysiology of the syndrome and evaluated their expression profiles in a well-characterized population of women with PCOS. Increased expression of *miR-21-5p*, *miR-23a-3p*, and *miR-26a-5p* and lower expression of *miR-103a-3p*, *miR-376a-3p*, *miR-19b-3p* and *miR-222-3p* were observed in PCOS, compared to control women. The differential expression of miRNAs has been suggested to target genes and to modulate pathways previously described as being part of PCOS physiopathology and its related metabolic disturbances [18,19,34,35].

Previous studies have also found abnormal expression patterns of circulating *miR-21*, *miR-23a-3p*, *miR-19b* and *miR-222* in women with PCOS [23,36,37]. Regarding *miR-21*, the results were conflicting. Sorensen et al. [38] found hypoexpression of this miRNA in the serum of women with PCOS compared to healthy women, whereas Jiang et al. [39] and Butler et al. [37] found it to be overexpressed. However, the first study evaluated *miR-21-3p*, whereas the latter two studies evaluated *miR-21-5p*. In our samples, although the differential expression of *miR-21-3p* was not statistically significant, this miRNA showed hypoexpression in the serum of women with PCOS compared to controls, while *miR-21-5p* showed hyperexpression, corroborating those studies. Another study [35] found no difference in serum *miR-21* expression between women with PCOS and healthy control women. However, that study did not specify which *miR-21* was assessed.

In contrast with our results, Xiong et al. [36] found downregulation of circulating *miR-23a-3p* in PCOS, and that it was positively associated with BMI. However, in agreement with our findings, there was no association of this molecule with androgen or gonadotrophin levels. Serum *miR-19b-3*p and *miR-222-3p* were also previously studied in women with PCOS, and both displayed increased expression compared to age- and BMI-matched healthy controls [20,37]. Although our study found that these miRNAs were downregulated in PCOS, we agree with the conclusions presented in previous reports that these molecules are not related to BMI, androgen or AMH levels in PCOS.

*miR-21-5p*, *miR-103a-3p* and *miR-376a-3p* were positively correlated with androgen levels in our PCOS population. Murri et al. [40] also demonstrated a potential role of testosterone concentration in the positive regulation of *miR-21-5p* and *miR-103*. In the work of Naji et al. [35], decreased levels of *miR-21-5p* were found in the serum of women with hyperandrogenic PCOS, although the difference was not significant, whereas Butler et al. [37] found no relationship between androgen levels and this miRNA. *In silico* analysis of *miR-21-5p* demonstrates that it may be involved both in steroid biosynthesis and in androgen receptor signaling pathways. The conflicting findings reported in these studies may be related to the different roles *miR-21-5p* may play in the manifestation of hyperandrogenism. In agreement with our results, *miR-103* has been previously positively correlated with testosterone levels [40] in PCOS, whereas to the best of our knowledge, our study is the first to demonstrate this correlation for *miR-376a-3p*. Interestingly, both of these miRNAs target the cyclin-dependent kinase 2 (CDK2) gene, which has been associated with phosphorylation of the androgen receptor [41].

Additionally, in women with type 2 diabetes, a condition related to PCOS [9,10], circulating *miR-23a* was suggested as a potential marker of this condition [42], whereas an increase in *miR-21-5p* was associated with the severity of the disease as well as with related vascular dysfunctions such as retinopathy and diabetic nephropathy [43,44]. This may be justified based on this miRNA role in angiogenesis, that culminate with the PI3K-AKT [45] signaling pathway, dysregulation, which is common in women with PCOS and insulin resistance [46]. Furthermore, another study reported a decrease in *miR-21-5p* in vascular smooth muscle cells after metformin treatment under diabetic conditions. Similarly, metformin treatment was also associated with decreased expression of *miR-26a*-5p and *miR-222-3p* in pancreatic stem cells and arterial mammary arteries of women with type 2 diabetes, respectively [47,48]. Furthermore, *miR-222-3p* levels were positively correlated with fasting glucose and with glucose 2 hours after OGTT in prediabetic patients, suggesting its potential use as a new biomarker for this condition [49]. Our *in silico* analysis showed that, together with *miR-21-5p*, *miR-26a-5p* and *miR-222-3p* targeted the PTEN gene, suggesting that the PI3K-AKT insulin signaling pathway should be further explored as a potential therapeutic target for insulin resistance in PCOS.

Other studies of populations of women with PCOS sought to assess the relationship between miRNAs and insulin resistance in the syndrome. Jiang et al. [50] found that *miR-122*, *miR-194*, and *miR-193b* were differentially expressed in glucose-tolerant and glucose-intolerant women with PCOS. In this study, these miRNAs were related to HOMA-IR. Increased circulating concentrations of *miR-122* are also strongly associated with the risk of developing type 2 diabetes mellitus in the general population [51]. Song et al. [52] found that *miR-6767* is downregulated in the serum of South Korean women with PCOS compared to healthy controls. This miRNA correlated positively with SHBG concentrations and negatively with glucose concentrations. In this current work, we did not find any correlation between the miRNAs studied and HOMA-IR or serum glucose levels.

The use of HOMA-IR instead of hyperinsulinemic euglycemic clamps, which are considered the gold standard for the diagnosis of insulin resistance, may have been a limiting factor in our study in the evaluation of the relationship between the miRNAs identified in this work and PCOS. However, HOMA-IR is the model most commonly used in clinical practice to assess insulin resistance, and the results obtained using this model show a good correlation with the standard [53]. In addition, most studies conducted in women with PCOS have used HOMA-IR to assess insulin resistance, making our results comparable [54].

Although in the current study we did not find significant correlations between most of the miRNAs studied and the main hormonal and metabolic features of PCOS, *miR-21-5p*, *miR-23a-3p*, *miR-222-3p*, and *miR-376a-3p* are highly expressed in ovarian tissue [55–59] and are associated with biological functions and signaling pathways that participate in the pathophysiological processes associated with the syndrome. *miR-21-5p* was originally identified as an LH/hCG regulatory gene in a murine model of ovarian granulosa cells [60], in which it was shown to promote follicular survival during ovulation. Mase et al. [61] found that silencing *miR-21-5p* reduced ovulation rates. Another study found *miR-21-5p* to be an important regulator of granulosa cell apoptosis and proliferation in PCOS [59]. Moreover, *miR-21-5p* was not identified in ovarian theca cells or in oocytes [62,63], suggesting its specific action in the granulosa cells or other cumulus cells [64]. *miR-376a-3p* was also identified in granulosa cells in murine models, and associated with primordial follicular development and granulosa cell proliferation [56], whereas *miR-23a-3p* promoted follicular granulosa cell apoptosis [58]. On the other hand, *miR-222-3p* was found to promote the progression of PCOS [57].

PCOS ovaries display a disordered folliculogenesis, but the underlying mechanisms are not clear yet [65–68]. Signaling pathways, as MAPK, FOXO, PI3K-AKT, mTOR, SMAD, and TGFß [46,69–72], may be involved in this process. In the prediction of biological processes using bioinformatics tools in our study, the molecular functions and signaling pathways related to miR-21-5p, miR-23a-3p, miR-222-3p and miR-376a-3p included functions and pathways involved in the physiology of folliculogenesis as well as in the processes of cell proliferation and apoptosis. In one sense, our results may bring insights into the mechanisms of this phenomenon. However, the manner through those miRNAs regulates those pathways during abnormal folliculogenesis in PCOS requires further investigation that is beyond the scope of the present study.

Despite the limitations of this study, we hypothesize that the differential expression of *miR-21-5p*, *miR-23a-3p*, *miR-222-3p* and *miR-376a-3p* found in the serum of women with PCOS may be related to the dysfunctional proliferation of granulosa cells, especially at more advanced stages of follicular development, or to the proliferation of other cumulus cells and ovarian vascular smooth muscle cells. The identification of miRNAs in blood and other cellular fluids is relatively recent and has stimulated the development of research with two objectives: first, to determine whether these molecules are useful as biomarkers of disease and, second, to determine whether these molecules serve as a previously unknown form of intercellular and intertissue communication [73].

In summary, our preliminary study demonstrated that *miR-21-5p*, *miR-23a-3p and miR-26a-5p* were upregulated and that *miR-103a-3p*, *miR-376a-3p*, *miR-19b-3p* and *miR-222-3p* were downregulated in serum samples obtained from a well-characterized cohort of women with PCOS compared to healthy normo-ovulatory controls. We also demonstrated that serum levels of *miR-21-5p*, *miR-103a-3p* and *miR-376a-3p* are positively correlated with androgen levels in the PCOS population. These miRNAs, in combination, are related to pathways involved in insulin signaling, steroids and endothelial regulation as well as in folliculogenesis. Our study may open a new line of investigation into the role of these regulatory molecules in PCOS and in its metabolic and reproductive consequences. Furthermore, it may also bring insights into possible therapeutic tolls based on molecular strategies.

## Supporting information

S1 Dataset (XLSX)
