# Correction: Evaluation of circulating microRNA profiles in Brazilian women with polycystic ovary syndrome: A preliminary study

**DOI:** 10.1371/journal.pone.0317139

**Published:** 2025-01-03

**Authors:** Giovana De Nardo Maffazioli, Edmund Chada Baracat, José Maria Soares, Kátia Cândido Carvalho, Gustavo Arantes Rosa Maciel

The third author’s name is spelled incorrectly. The correct name is: José Maria Soares Jr.. The correct citation is: De Nardo Maffazioli G, Baracat EC, Soares Jr. JM, Carvalho KC, Maciel GAR (2022) Evaluation of circulating microRNA profiles in Brazilian women with polycystic ovary syndrome: A preliminary study. PLOS ONE 17(10): e0275031. https://doi.org/10.1371/journal.pone.0275031

## References

[1] De Nardo Maffazioli G, Baracat EC, Soares JM, Carvalho KC, Maciel GAR (2022) Evaluation of circulating microRNA profiles in Brazilian women with polycystic ovary syndrome: A preliminary study. PLOS ONE 17(10): e0275031. 10.1371/journal.pone.0275031 36206272 PMC9543946

